# Modern Surgery

**Published:** 1894-05-05

**Authors:** 


### The Hospital

An Institutional Journal of

The Medical Sciences and Hospital Administration, with

AN EXTRA NURSING SUPPLEMENT.

Vol. XVI.—No. 397.] [May 5, 1894.

### Modern Surgery.

The May number of the *Nineteenth Century* contains an interesting and instructive article, by Mr. Percy Dunn, on "Modern Surgery," an article which is well-calculated to direct public attention to the extent and character of the benefits which the science and art referred to have conferred upon mankind. At the present day, when many endeavours are made to minimise these benefits, and to cast doubt upon the value and importance of the experimental methods by which, in most cases, the power to confer them has been gained, an article of this description is extremely well-timed; more especially as one of the most conspicuous wants of the medical profession is that of some recognised channel through which, without diminution of self-respect or lowering of professional status, its members may speak to the public, and may throw the light of special knowledge upon many of the social and other problems of the day. As matters now stand, the magazine article savours somewhat too much of personal advertisement to find favour with practitioners of eminence; and yet how many problems might be rendered more intelligible by its means? We do not in the least desire to claim a monopoly of knowledge, on any matters lying beyond their daily work, for members of the medical profession; but there are subjects upon which their opinions would not only be worthy of consideration, but would be helpful in dispelling much prevailing ignorance. It is manifest, for example, that all successful education must be so directed as to be in harmony with the physiological processes through which the mental faculties arrive at the full measure of their growth and efficiency; and yet how few schoolmasters there are who have any further conception of the meaning of the word "physiology" than such as they may obtain from a dictionary. The whole field of ethics, moreover, appears under a new aspect when the influences of structure and of heredity have been examined, and when the significant fact that a man will be found habitually repeating some trick or gesture which was characteristic of his grandfather, or of a still more remote ancestor, has been looked at in the light of its true meaning and importance. On these, and many kindred questions, medicine has still to take up her parable and to teach; and the coming of the day when she can teach effectively is likely to be hastened in proportion to the degree in which her achievements in her own peculiar domain are understood by the half educated community. If the vulgar are to accept the conclusions of philosophers on matters beyond their own capacity, they must first see these conclusions justified on such as are within the limits of their experience.

It is, we think, to be regretted that Mr. Dunn has limited the scope of his article by the use of the word "surgery," when he might so easily have said "medicine," and have included surgery within its limits. In truth, the two branches of the healing art have advanced together, and by well-nigh equal strides; but it is the fortune of surgery, in the narrow sense of the word, to appeal more strongly than medicine to the senses, and hence more vividly to impress the imagination. On this ground, and on the basis of the most profound ignorance of the real position of either, we may constantly hear the average dunce endeavouring to extol surgery at the expense of medicine, and talking of the latter as if it were still in the stage of growth which has been immortalised by Molière. Mr. Dunn makes much of the illustration, and it is a good one, that the time required for recovery from a great amputation was formerly six weeks, and is now a fortnight, in addition to the fact that the operation, which was formerly attended by a large mortality, is now almost invariably followed by recovery. This is very true; but we must not forget the long agony and the frequent heart disease which were entailed, only a few years ago, upon the sufferers from acute rheumatism. Dr. Baillie was credited with the saying that the only thing good for acute rheumatism was "six weeks;" but now, under the influence of modern improvements in treatment, it has been converted into what may almost be described as a trivial disorder, in which the heart complications, almost invariably sooner or later fatal when they arise, are scarcely any longer to be feared. Surely, the improved methods of performing and treating amputation, and the improved methods of treating rheumatism, have at least an equal claim upon us for grateful recognition; even if the rheumatism, by reason of the much greater frequency of its occurrence, be not entitled to take the first place-

Mr. Dunn will probably say that he speaks and writes as a surgeon, not as a physician, and that he therefore confines himself to the topics which are most familiar to him; but this consideration cannot entirely justify his neglect of the sister art to his own. Even the Legislature has been more enlightened, since it will no longer permit entrance into the medical profession to anyone who does not possess a competent acquaintance with all its branches. A few years ago, it was possible to obtain a licence in surgery without a knowledge of medicine, or a licence in medicine without a knowledge of surgery; but no one is now eligible to receive any kind of qualification, or to be placed upon the Medical Register as a member of the profession, until he has passed examinations in the three branches of medicine, surgery, and midwifery. The very fact of the change is calculated to educate the public into some conception of the absolute interdependence and essential similarity of the three.

Mr. Dunn writes easily and well, and his article will be read with pleasure and profit, not only by the public, but even by members of his own calling. He has no very new facts to bring forward; the truth being that no very important progress, of such a character as to admit of ready explanation to the unskilled, has been made during the last four or five years. He mentions antiseptic surgery generally, and dwells especially upon the surgery of the brain, the spinal cord, the eye, and the abdominal cavity. A good deal of his ground was covered by an article under the same title, which appeared in the *Times* some years ago, and still more in one of the essays contained in a comprehensive work, edited by Mr. Humphry Ward and published in the Jubilee year, which was entitled "The Reign of Victoria." It is to be wished that Mr. Dunn had made a greater endeavour to supply deficiencies in the writings of those who have preceded him on the same road; and there are certainly two directions in which he might have done this with advantage. He might have shown the enormous benefits which have been conferred upon mankind by experimentation on the lower animals (to which, for example, all the successes of brain and spinal surgery are directly attributable), and he might have shown how prodigiously greater and more important has been the share of this country, in making the advances which he records, than that not only of any other, but of all others put together. England is not more absolutely and unquestionably the mother of railroads than she is the mother of surgery; and the fact is one which her instructed children ought not to suffer to fall into oblivion. Just as much as when Mr. Muntle called himself "Mantalini," it is still the fashion among some of the silliest members of our people to extol the medical and surgical devices of foreigners, and to go to other countries for the assistance which they could obtain far better at home. The fashion is largely maintained by the systems of indirect puffery which are strenuously kept in operation by syndicates of hotel-keepers at continental watering places, and it scarcely ever takes the English tourist to the men of real professional eminence in the countries which he visits in search of cure. The essay in "The Reign of Victoria" just touches upon this branch of the subject, and points out that, while in preventive medicine we stand alone, the three "epoch making" discoveries in relation to our art, the discovery of the circulation, of the functions of the spinal cord, and of reflex action, were all English. Antiseptic surgery is English. Anæsthesia in surgery is American, and it may be doubted whether the whole of the European contributions to the general progress of the art have not been surpassed, in value to the human race, in the relief of suffering, and in the preservation of life, by the one English achievement of the successful removal of ovarian tumours, which, in the hands of two of our countrymen alone without considering the hundreds of surgeons who have followed their teaching, is said to have added nearly fifty thousand years to the life of womanhood. If Mr. Dunn should write more upon surgery we trust that he will seek to assert the claims of Great Britain to have brought about the greater part of the progress which he has so pleasantly and so instructively recorded.

